# Rapid isolation of extracellular vesicles from stem cell conditioned medium using osmosis-driven filtration

**DOI:** 10.1080/14686996.2025.2485668

**Published:** 2025-04-03

**Authors:** Casey Y. Huang, Helen Nguyen, David J. Lundy, James. J Lai

**Affiliations:** a Department of Material Science and Engineering, National Taiwan University of Science and Technology, Taipei, Taiwan; b Graduate Institute of Biomedical Materials and Tissue Engineering, Taipei Medical University, Taipei, Taiwan; c International PhD, Program in Biomedical Engineering, Taipei Medical University, Taipei, Taiwan; d Center for Cell Therapy, Taipei Medical University Hospital, Taipei, Taiwan; e Department of Bioengineering, University of Washington, Seattle, WA, USA

**Keywords:** Exosome, mesenchymal stromal cell, cardiomyocyte, bioprocessing, polymer

## Abstract

Extracellular vesicles (EVs) hold significant promise as biomarkers and therapeutics, yet their isolation remains challenging due to their low abundance and complex sample matrices. Here, we introduce EV-Osmoprocessor (EVOs), a novel device that leverages osmosis-driven filtration for rapid and efficient EV isolation. EVOs employs a high osmolarity polymer solution to concentrate EVs while simultaneously removing smaller contaminants. Compared to traditional methods such as ultracentrifugation and precipitation, EVOs offers speed and convenience, achieving a 50-fold volume reduction in under 2 h. Our results show that EVOs retained EVs and removed >99% albumin from the cell conditioned culture medium (CCM). The isolated EVs exhibited a particle size distribution centered around 140 nm, which was very similar to EVs isolated via precipitation or ultracentrifugation. The standalone EVOs process achieved a particle:protein ratio (EV purity) of ~10^7^ particles/µg protein. Comprehensive characterization, including cryo-electron microscopy, validation of protein markers and known miRNA cargo confirmed the successful isolation of EVs. Functional assays, based on protection of cardiomyocytes from hypoxia/reoxygenation injury, demonstrated the bioactivity of EVOs-isolated EVs. Furthermore, we show that EVOs can be used to concentrate 30 ml of CCM into a 0.5 ml solution, which was then further processed with size-exclusion chromatography (SEC), improving EV purity to ~10^9^ particles/µg protein. This work establishes EVOs as a promising tool for EV research and clinical applications, offering a streamlined approach to EV isolation with enhanced analytical performance.

## IMPACT STATEMENT

EVOs, an osmosis-driven filtration device, rapidly isolates extracellular vesicles with high yield and preserved cargo and functionality, offering a scalable, efficient solution for EV research in under 2 h.

## GRAPHICAL ABSTRACT

**Figure:**
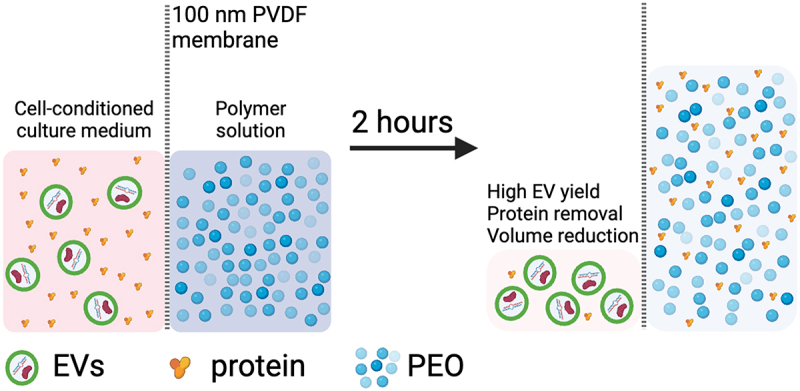

## 1. Introduction

Extracellular vesicles (EVs) are cell-secreted membrane-bound particles with diameters of 50 to 1,000 nm [1]. Their composition of proteins, lipids, carbohydrates and nucleic acids facilitates intracellular paracrine and endocrine communication [2,3]. EVs are present in several body fluids, including blood, urine, milk and saliva, and can be isolated from the cell conditioned culture medium (CCM) [4–6]. The small EV population of 50 to 200 nm (often referred to as ‘exosomes’) have received considerable research attention for use as therapeutics, drug delivery vehicles, and biomarkers [7,8].

One challenge in the field is to separate EVs from the medium in which they are contained at sufficient yields and purity [9]. Obtaining EV isolates of sufficient purity is important for quality control, and for downstream applications in diagnostics, biomarker research or potential therapeutic uses [10]. In particular, low-purity EV isolates may underestimate the true EV dose and confound results with excess non-EV proteins. Common EV isolation methods include ultracentrifugation (UC), ultrafiltration, precipitation, immunoaffinity capture, and size-exclusion chromatography (SEC) [7]. However, each technique has trade-offs in terms of processing speed, throughput, ease-of-use and their ability to separate EVs from non-EV proteins and particles [9,11–14]. Optimal isolation techniques vary between source materials. For example, the concentrations of EVs and non-EV proteins in blood or CCM vary by orders of magnitude [15–17]. UC and ultrafiltration are commonly used to isolate EVs from CCM since they have a relatively high throughput [7,14]. UC can produce relatively pure EV isolates, which are suitable for research use and pre-clinical testing, but it is time-consuming, requires personnel training, and there is intra-batch and intra-operator variability from washing and re-suspending EV pellets [18,19]. Ultrafiltration utilizes hydrostatic pressure to force fluid through membranes with ~100 nm diameter pores, rapidly isolating EVs [12]. However, the high pressure applied during filtration can damage EVs through shear forces, and the EV yield can be affected by membrane adhesion and blockage [20]. Another approach, SEC, uses chromatography columns to separate EVs from smaller free proteins, producing high-purity EV isolates. However, the overall loading capacity is limited and the fraction collection process dilutes the EVs in the column outflow, requiring additional concentration steps [11,21]. Lastly, precipitation methods involve the use of excluded volume hydrophilic polymers such as polyethylene glycol (PEG) [13]. These polymers attract water molecules, causing phase separation of the less soluble components, including but not limited to EVs [22]. These methods are reproducible and easy to follow, but produce lower purity EV isolates which also contain residual polymer. The polymer composition of most commercial products is also proprietary, and may affect downstream assays [22].

**Scheme 1.**
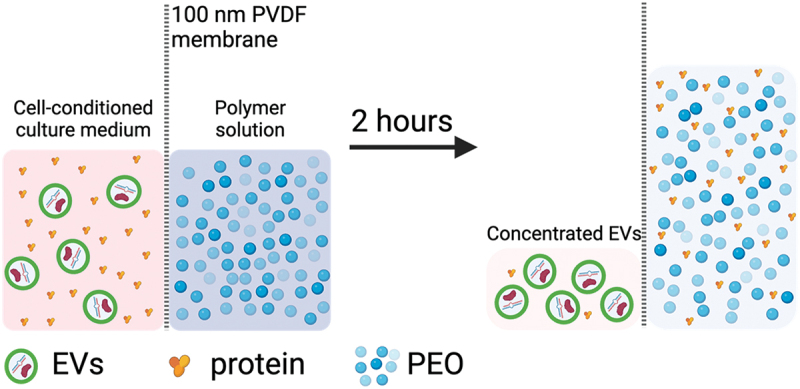
Schematic showing extracellular vesicle (EV) isolation from cell conditioned medium (CCM) via osmosis-driven filtration through a porous membrane. After two hours, background proteins are removed and the CCM volume is reduced, thus concentrating the EVs in the CCM specimen.

Previously, Chen and colleagues utilized osmosis-driven filtration to concentrate the SARS-CoV-2 nucleocapsid (N) protein nearly 200-fold [23]. Selecting a membrane with a suitable molecular weight cutoff allowed for retention of the N protein, while an osmotic gradient removed water, reducing the total specimen volume. We hypothesized that this approach could be adapted to isolate EVs present in CCM while simultaneously removing water and medium molecules smaller than the membrane pores (Scheme 1). We aimed to develop a process that would be rapid while requiring minimal user input to produce reproducible results. To test this hypothesis, we designed a device, EV-Osmoprocessor (EVOs), where samples could be easily and repeatably subjected to osmosis-driven filtration. We then characterized the device performance using model specimens of proteins and specific sizes of nanoparticles, followed by more complex biological samples. The International Society of Extracellular Vesicles (ISEV) provides recommendations for EV characterization, including measuring particle count and protein concentration, verifying lipid bilayer morphology, validating cargo/surface markers, confirming miRNA cargo and confirming expected functions [1,24]. Thus, we followed these guidelines, and compared isolation yield, purity and function to EVs isolated by UC or precipitation methods. The mesenchymal stem/stromal cell (MSC) secretome, including EVs, has a wide variety of immunomodulatory, pro-angiogenic and anti-apoptotic functions, and has shown good promise as therapeutics following myocardial infarction [25]. Much of the MSC-EV activity has been attributed to the protection of cardiomyocytes by EV micro-RNA (miRNA) cargo, including miR-21 and miR-125b [26–28]. Therefore, we validated the presence of known cardioprotective miRNAs and used MSC-EVs in a human cardiomyocyte hypoxia/re-oxygenation injury model to evaluate the functional performance of EVs isolated by EVOs.

## 2. Methods

### 2.1. Device fabrication

**Scheme 2.**
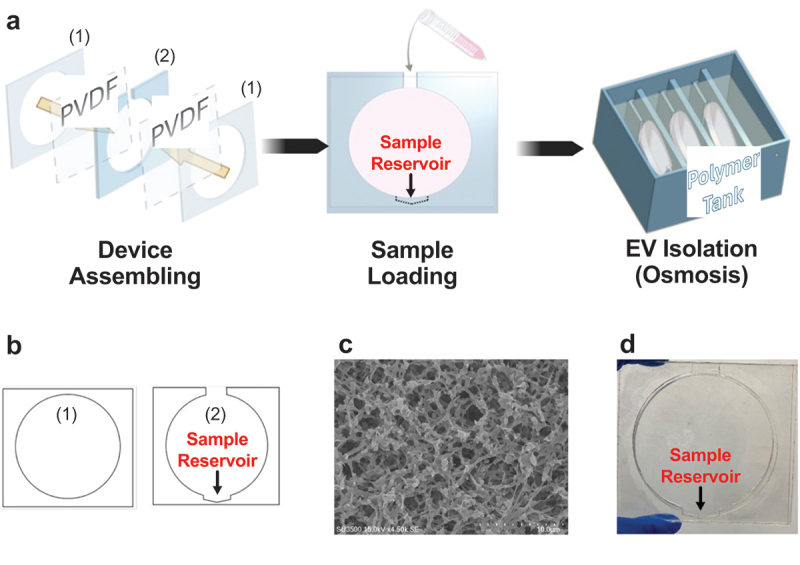
(a). The workflow starts from assembling the device. The device is fabricated by securing 100 nm PVDF membranes between the laser-cut acrylic sheets to form the sample holder. Once the CCM is introduced (sample loading), the device is inserted into the polymer tank to start the EV isolation/osmosis. Multiple devices can be used simultaneously to increase the processing capacity; (b). The CAD designs for the device acrylic components. (1) shows the two outer layers and (2) illustrates the middle spacer including a 200 µl sample reservoir which is not subjected to osmosis, thus retaining the EVs; (c). A representative SEM image of the PVDF membrane; (d). A photo of the assembled device.

The device (Scheme 2) was designed using Adobe Illustrator. The sample holder was constructed from a 1.5 mm thick poly(methyl methacrylate) board (Woodmall), cut to size using a laser cutter. The center part of the device contains a 200 µl reservoir which does not contact the filtration membrane. Each sample holder measures 93 mm × 85 mm. The final device was assembled by sandwiching PVDF membranes with 100 nm pores (Millipore) between two laser cut layers using double coated PET tape (3 M) and super glue (Gorilla Glue). The device reservoir was 3D printed using a da Vinci Super 3D (XYZ printing, TAIWAN).

### 2.2. EVOs process

The polymer tank was filled with 0.5 g/ml 100 kDa polyethylene oxide, PEO (Sigma-Aldrich). Ten milliliters of specimen was loaded into each sample holder, then placed into the tank (Scheme 2). After osmosis (<2 h), the 200 µl of retained solution was collected. To test PEO permeation, 10 ml of ddH_2_O was loaded into the sample holder and subjected to osmosis. The retained 200 µl was lyophilized and weighed. Water and the original PEO solution were used as controls.

### 2.3. Precipitation

ExoQuick-TC (System Biosciences) was used, following the manufacturer’s instructions. Briefly, 2 ml reagent was added to 10 ml CCM, mixed and incubated overnight at 4°C. The solution was centrifuged at 1,500 *g* for 30 min, supernatant removed, then centrifuged at 1,500×*g* for 5 min. The resulting pellet was resuspended in 200 µl of sterile PBS.

### 2.4. Ultracentrifugation

A standardized differential centrifugation method was followed [29]. CCM was centrifuged at 500×*g* for 10 min, then 3000×*g* for 20 min at 4°C to remove cells and debris. Supernatant was filtered through a 0.22 µm membrane then ultracentrifuged (Optima XE-90, Beckman Coulter, USA) at 100,000×*g* for 16 h at 4°C. Supernatant was removed and the pellet was washed with PBS then centrifuged at 100,000×*g* at 4°C for 1 h. Resulting EVs (from the initial 10 ml CCM) were then re-suspended in 200 µl PBS.

### 2.5. Nanoparticle permeation and quantification

Fluorescent polystyrene nanoparticles of 20, 100 and 200 nm diameter (FluoSpheres, Thermo Fisher Scientific) were diluted in sterile PBS to final concentrations of 50 µg/ml (20 nm particles) or 8 µg/ml (100 and 200 nm particles). Following processing by EVOs, the nanoparticles were quantified using a published method [30]. Briefly, xylene (reagent grade, Sigma-Aldrich) was added (1:1 v/v), and the solution was sonicated and freeze-thawed to lyse the nanoparticles. The mixture was centrifuged (13,000×*g* for 30 min), and the supernatant was measured using a spectrofluorometer at excitation/emission wavelengths of 505/515 nm, respectively. Samples were quantified using a standard curve prepared from stock solutions of each nanoparticle processed by the same method.

### 2.6. Osmosis kinetics and osmotic pressure measurement

Ten milliliters of PBS or CCM were processed to assess the solute removal rate. The retained volume inside the device was measured at 15-min intervals for 1 hour. The osmotic pressure was measured using a micro-osmometer (Advanced Instruments) at Academia Sinica Neuroscience Core Facility (ASNCF).

### 2.7. SDS-PAGE

Samples were mixed with 4 × Laemmli buffer (Bio-Rad), boiled at 95°C for 5 min, then placed on ice. Electrophoresis was performed at 100 V using 4–20% Mini-PROTEAN® TGX™ precast protein gels (Bio-Rad). After electrophoresis, the gel was washed three times with DI water, stained with Bio-Safe™ Coomassie Stain (Bio-Rad) for 1 hour at room temperature, then destained with DI water for 1 hour before imaging.

### 2.8. Determination of total protein and albumin concentrations

Total protein concentrations were determined by the BCA protein assay kit (Thermo Fisher Scientific). Bovine Serum Albumin (BSA) (Sigma-Aldrich) was measured by ELISA. BSA specimens were coated onto Nunc-Immuno™ Maxisorp 96-well plates (Thermo Fisher Scientific) overnight at 4°C in 0.1 M Na_2_CO_3_, pH 9.6, and then the plate was blocked with SuperBlock® PBS for 30 min at room temperature. Anti-BSA antibody (GTX29092, GeneTex) was added to the wells for 1 hour incubation at room temperature. Then, HRP conjugated goat anti-mouse IgG (31430, Invitrogen) was diluted 1:2000 in 10 mm PBS-T and added to the wells for 1 hour incubation at room temperature. Color was developed by adding TMB (Pierce 1-Step Turbo TMB, Thermo Fisher Scientific) for ca. 10 min at room temperature and stopped by adding 1 M HCl. The absorbance was recorded with a Tecan Spark® microplate reader (Tecan, Switzerland) at 450 nm with a background of 650 nm. Between each incubation, the wells were washed five times (300 μl/well) with PBST. Samples were quantified using a calibration curve constructed using BSA standards.

### 2.9. Human mesenchymal stromal cell culture

Human bone marrow mesenchymal stromal cells (BM-MSCs) were purchased from Lonza and routinely cultured in DMEM with 10% (v/v) FBS in 5% CO_2_ at 37°C. To generate CCM, a basal medium for conditioning was prepared by substituting regular FBS for commercial EV-depleted FBS (Thermo, A2720801). We have previously validated that this FBS is >99% EV depleted [29]. BM-MSCs at 80% confluence were used to condition the medium. Basal medium (i.e. without cell conditioning) was used as a control.

### 2.10. Nanoparticle tracking analysis (NTA)

Particle concentration and size were measured by NanoSight NS300 (Malvern, United Kingdom) nanoparticle tracking analysis (NTA). Samples were diluted to obtain 20–200 particles/frame and recorded for 60 seconds. Particle counts are reported based on the original sample volume.

### 2.11. Cryo transmission electron microscopy (cryoEM)

Samples were prepared and imaged by technicians at the Academia Sinica Cryo Electron Microscope Core Facility (ASCEM), Taipei, Taiwan. Samples were diluted ten-fold, and 10 µl of each solution was vitrified using a FEI Vitrobot (Thermo Fisher Scientific, USA) on plasma-treated carbon grids, and images were captured using a Tecnai G2 F20 (Thermo Fisher Scientific, USA).

### 2.12. Cardiomyocyte culture and hypoxia/reoxygenation injury model

AC16 human cardiomyocytes (Merck, SCC109) were routinely cultured in DMEM/F12 with 12.5% (v/v) FBS in 5% CO_2_ at 37°C. To establish the hypoxia/reoxygenation (H/R) model, cells were seeded in a 96-well plate (4,000 cells/well). After 24 h in normal conditions, the medium was exchanged to include EVs or the same volume of PBS as a vehicle control. EV doses were normalized by particle count (1.2 × 10^5^ particles per cell). To simulate myocardial infarction, the cells were subjected to 48 h hypoxia using airtight boxes with anaerobic sachets (AnaeroPack), validated by oxygen-sensitive strips. Re-oxygenation injury was induced by returning plates to normoxia. Separate plates of cells were kept under routine conditions throughout as a normoxic control.

### 2.13. Cardiomyocyte viability and cell damage assessment

To assess cardiomyocyte injury after hypoxia, culture medium LDH leakage was measured using a kit (Dojindo CK12–20). One hundred-microliter culture medium was added to 100 µl LDH assay working solution and incubated for 30 min in the dark. Fifty-microliter stop solution was added, and the absorbance was read at 490 nm. Cell culture medium was then replaced with a fresh, complete medium. After 4 h of re-oxygenation, cardiomyocyte dehydrogenase metabolic activity was measured by CCK-8 assay (Dojindo, CK04), using normoxic cells as the baseline for maximum activity. Absorbance values of blank samples (i.e. culture medium without cells) were subtracted for both LDH and CCK-8 assays.

### 2.14. RT-qPCR

miRNA was isolated by using a kit (Qiagen 217,084). cDNA synthesis from miRNA was performed using a miRCURY LNA RT Kit (Qiagen 339,340). Twenty nanogram RNA extract was added to 20 µl cDNA synthesis reaction. qPCR was run by using miRCURY LNA SYBR Green PCR Kit (Qiagen 339,347) on a StepOne Real-Time PCR System (Thermo Fisher Scientific, USA) for the primers listed in Supplementary Table S1. Two-microliter cDNA template was added to each well, with 100 nM primers in reaction volume of 10 µl. StepOne v2.3 software was used to collect and analyze the amplification of miRNA. For each primer set, samples without cDNA templates were run as a negative control.

### 2.15. Size exclusion chromatography

CCM was first concentrated by EVOs as described above. Size-exclusion chromatography columns (Izon qEV original 35 nm Gen 2, 0.5 ml) were washed and equilibrated following the manufacturer's protocol. 0.5 ml of CCM was loaded per column and 0.5 µl fractions were collected. The first 4 fractions (flow-through) were discarded. Subsequent EV fractions were collected and measured by NTA and BCA, as described previously with fractions 5–7 selected as EV-containing fractions [31,32].

### 2.16. Statistical analysis

Results were compiled in Microsoft Excel or Google Sheets. Statistical analyses were performed using GraphPad Prism 10. Specific statistical tests used are described in figure legends, and *p* values for each comparison are shown in the figures. Error bars in the figures show standard error of the mean unless otherwise stated, and individual data points are shown where available.

## 3. Results

### 3.1. Device fabrication and usage

A schematic illustration of the workflow is shown in Scheme 2a. The sample holder was assembled by layering PVDF membranes (100 nm pores) within three pre-cut acrylic sheets (Scheme 2b,c). The holder was designed with a 200 µl sample reservoir at the bottom for retaining the processed specimen (Scheme 2b,c). A 10 ml of specimen was introduced through the top opening, and the sample holder was then inserted into the polymer tank, containing the PEO solution, to initiate the osmosis process. When the specimen solution level was reduced to the sample reservoir, the osmosis process stopped spontaneously because the reservoir did not interface with the polymer solution. Upon completion of osmosis, the retained, processed solution was collected from the reservoir for further analysis.

### 3.2. Kinetics of sample volume reduction

**Figure 1.**
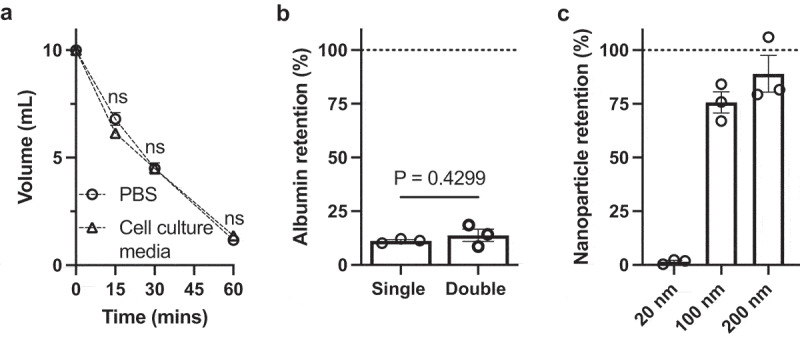
Kinetics and size-dependent removal of compounds via osmosis.

For initial proof-of-principle experiments, we used model specimens, including DI water, PBS, and CCM. For all experiments, 10 ml samples were reduced to 200 µl (50-fold volumetric reduction). Since the 100 kDa PEO chain is smaller than the 100 nm membrane pore size, some polymers may diffuse across the membrane into the specimen, which was evaluated using DI water specimens (Supplemental Figure S1). With a single filtration membrane sample holder, some residual PEO (≥60 mg) crossed into the specimen during osmosis, but the two layers of membrane significantly reduced PEO permeation to less than 15 mg. Therefore, sample holders with two layers of membrane were then used for following experiments. The process kinetics evaluation using both PBS and cell culture medium did not show difference in volume reduction rates, which were 8.83 and 8.63 ml per hour for PBS and CCM, respectively (Figure 1a). This is likely due to the large osmotic pressure gradient. Based on the osmolarity measurements for the PEO solutions (Supplementary Figure S2), we estimated the osmolarity for the 0.5 g/ml PEO solution was 2329 mOsm/l, which is >8-fold higher than both cell culture media and PBS [33].

a. 10 ml of PBS or cell culture medium (CCM) was processed using EVOs. The graph shows the volume remaining at 0, 15, 30 and 60 min. Dotted lines show interpolated readings. N = 3 samples per data point. CCM and PBS volumes at each time point were compared using repeated measures two-way ANOVA. ns = not significant (p > 0.05); b. Removal of spike-in albumin from 10 ml PBS processed using EVOs. An average of 88% of albumin was removed. Single and double membranes were compared by unpaired t-test, with the *p* value indicated; c. Solutions containing nanoparticles of 20, 100 or 200 nm diameter were processed using EVOs, and percentage retention was calculated. Each data point shows a separate experimental repeat. Error bars show the standard error of the mean.

### 3.3. Demonstrating size-dependent retention and removal using EVOs

EV isolation requires removal of non-EV proteins, of which albumin is the most abundant. To test this, a PBS solution with 20 µg/ml BSA was subjected to osmosis. The results (Figure 1b) show that EVOs processing removed more than 85% of the albumin, and the addition of a second membrane did not inhibit protein removal. Since the EV population of greatest interest are approximately 80–200 nm in diameter, we next examined size-dependent retention and removal using model samples containing 20, 100, or 200 nm diameter solid nanoparticles. The results showed that 1.5 ± 1.0% of 20 nm particles were retained (ca. 98.5% removal), whereas 75.6 ± 8.5% and 81.5 ± 14.8% of 100 and 200 nm particles, respectively, were retained (Figure 1c). Together, these data show that the EVOs retained EV-sized particles while removing smaller particles as well as albumin.

### 3.4. Processing of cell conditioned culture medium

**Figure 2.**
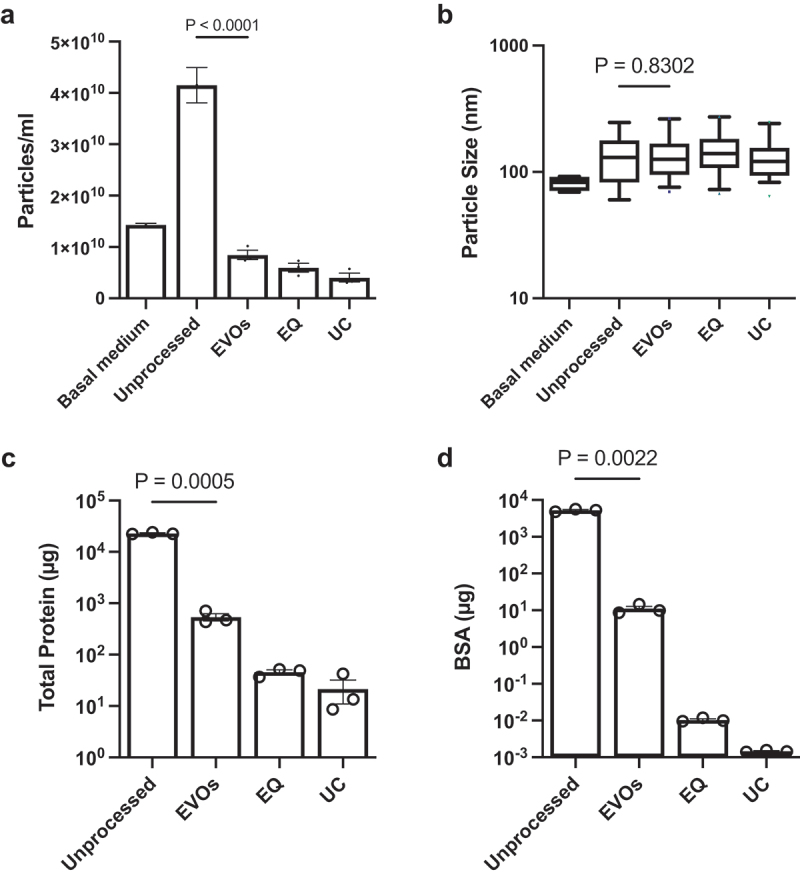
Isolation of extracellular vesicles from cell conditioned medium.

**Figure 3.**
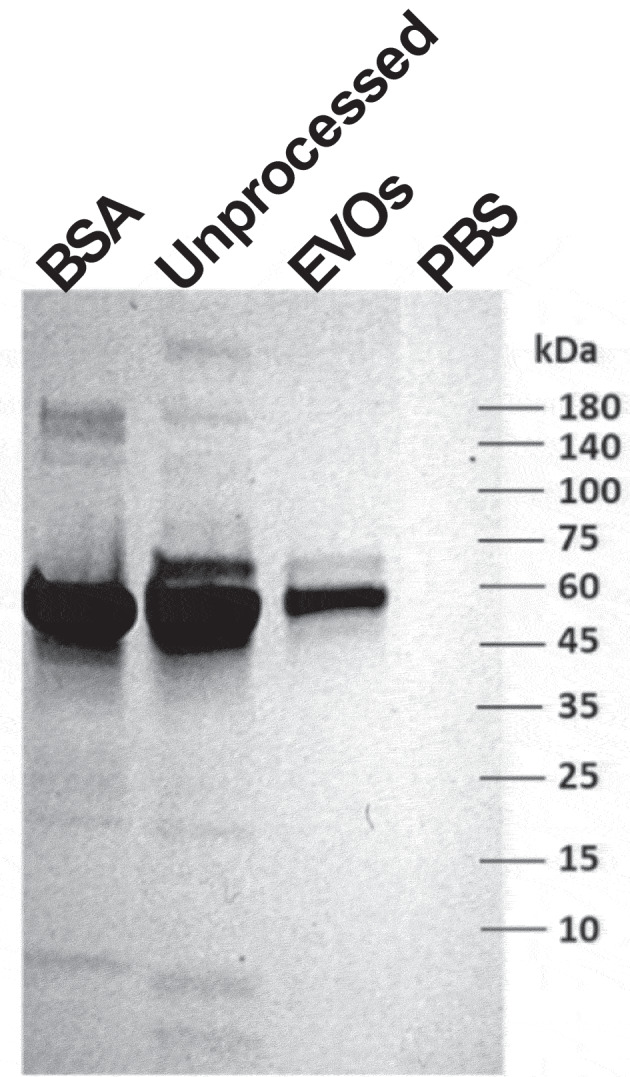
Protein composition of EVOs-processed cell conditioned medium.

Next, we evaluated whether EVOs were capable of processing CCM, which is a complex biological mixture containing EVs along with other cell products and components of the basal medium. We hypothesized that EVOs would retain EVs, while smaller proteins and particles would be removed. Additionally, we expected that the volume reduction should lead to EV concentration. To evaluate this, we compared unprocessed CCM against CCM processed using EVOs. We also used UC and a commercial precipitation reagent (ExoQuick, EQ) as two widely used benchmark methods to validate our isolations. For all methods, the same volume of CCM was processed, and isolated EVs were resuspended in 200 µl. Figure 2a shows the resulting particle concentrations measured by NTA. Basal medium contained 1.43 × 10^10^ particles/ml which increased to 4.15 × 10^10^ particles/ml after conditioning by MSCs. This increased in particle count is attributed to the MSC secretome. Compared to the unprocessed CCM, EVOs significantly reduced the particle count to ≤1 × 10^10^ particles/ml, as did UC and EQ. Figure 2b shows the particle size distributions measured by NTA. Basal medium had the smallest particles (81.8 ± 5.8 nm mean diameter), whereas the unprocessed CCM contained larger particles (135.4 ± 26.4 nm mean diameter). Specimens processed by all three methods exhibited similar particle size distributions and mean particle diameters, with no statistically significant differences. The processed specimens were also assayed for their total protein levels (Figure 2c) and a specific assay for BSA (Figure 2d). The total amount of protein after EVOs was 540 ± 151 µg (~97% reduction compared to unprocessed CCM, *p* = 0.0005), which was higher than after UC and EQ. The BSA ELISA results showed a similar trend with 99.7% of BSA removed by EVOs (*p* = 0.0022); however, UC and EQ were able to remove 99.9% albumin. Finally, the overall protein makeup of EVOs-processed samples was profiled using SDS-PAGE, as shown in Figure 3. Examination of the gel revealed multiple bands ranging from <10 kDa to >180kDa in the unprocessed CCM. Some bands were at the same position as BSA; this is expected since BSA is abundant in CCM. When comparing the unprocessed and EVOs-processed samples, the latter only exhibited a few bands between 45 to 75 kDa, including albumin. Together with the BCA and ELISA results, these data show that EVOs could remove most background proteins from CCM in less than 2 h.

a. Particle counts of basal (unconditioned) medium, unprocessed CCM, and CCM processed using osmosis (EVOs), precipitation (EQ) or ultracentrifugation (UC) methods. Error bars show an SD of n = 3–9 samples per group. b. Particle sizes of each preparation, determined by NTA. Bars show the 10–90th percentile and the line shows the median particle size. No significant differences were found between processed CCM specimens; c. Total protein amount in each preparation, measured by BCA protein assay. Unprocessed and EVOs were compared by Welch’s t test; D. Total albumin amount in each preparation, measured by ELISA. Unprocessed CCM and EVOs-processed CCM were compared by t-test. Each data point shows a separate experimental repeat. Error bars show standard error of the mean unless otherwise stated. *p* values for each statistical comparison are shown.

SDS-PAGE gel showing proteins found in basal medium, unprocessed CCM and CCM following processing with EVOs. PBS is shown as a background control and a sample of pure BSA is shown to identify albumin.

### 3.5. Confirmation of EV isolation

**Figure 4.**
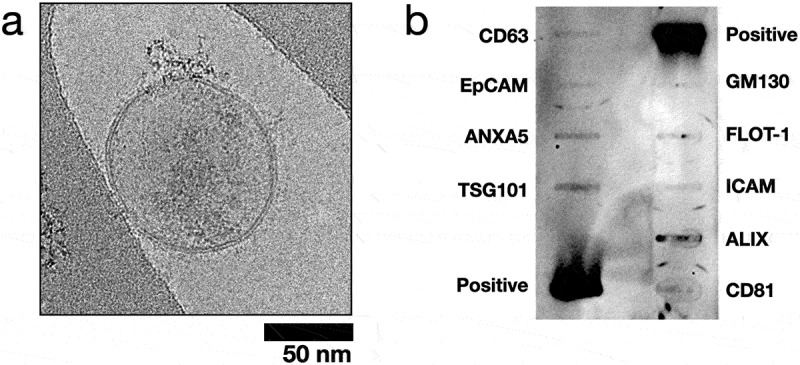
Validation of extracellular vesicle isolation by EVOs.

**Figure 5.**
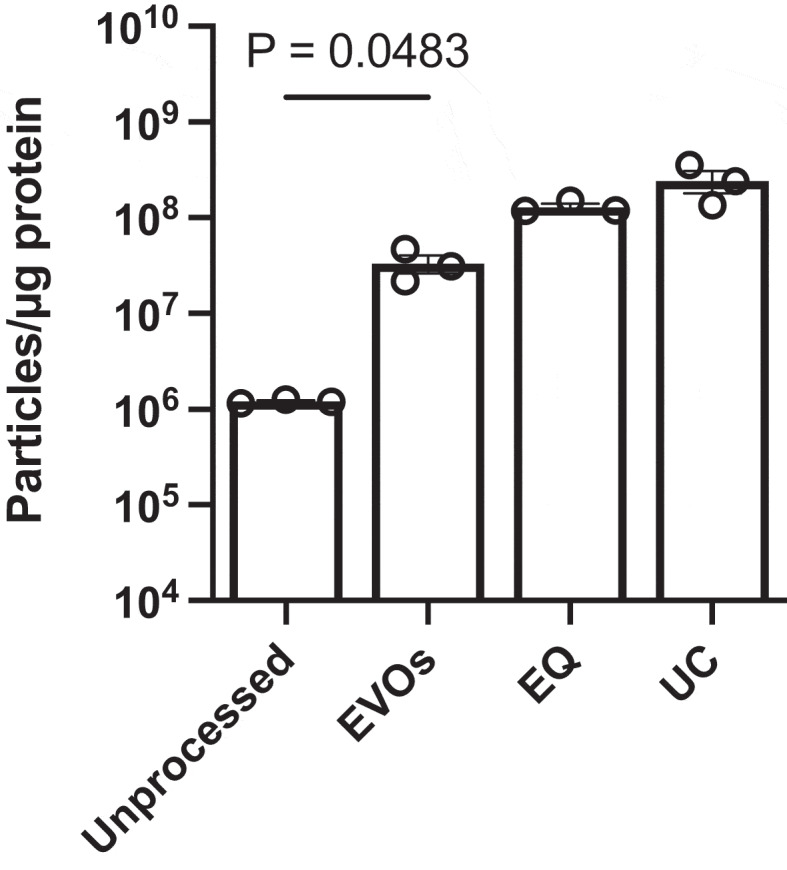
Purity of extracellular vesicles isolated by EVOs.

Since the prior assays cannot specifically distinguish EVs from non-EV particles/proteins, we assessed EVOs-processed CCM following the latest ISEV recommendations [1]. CryoEM was used to inspect the morphology of isolated EVs. Figure 4a shows a representative cryoEM image of specimens processed by EVOs. UC and EQ EVs are shown in Supplemental Figure S3. All samples contained spherical vesicles with lipid bilayer membranes and diameters of approximately 100 nm, confirming that the particles measured by NTA were indeed EVs rather than other non-EV particles such as protein aggregates or lipoproteins. To detect EV protein markers, we used an antibody-based membrane array (Figure 4b) which confirmed the presence of surface markers (CD81, CD63) and cargo/biogenesis markers (TSG101 and ALIX) in CCM samples processed using EVOs. Next, we measured the particle:protein ratio, which is a measurement of EV purity [34]. As shown in Figure 5, EVOs significantly increased EV purity to ca. 3.3 × 10^7^ particles/µg (*p* = 0.0483) compared to unprocessed medium, ca. 1.2 × 10^6^ particles/µg. However, the purity was not as high as EQ or UC which achieved ratios of 1.2–3.6 × 10^8^ particles/µg. Finally, MISEV guidelines recommend that cargo and functional activity of EVs should be confirmed in addition to morphology and protein markers. Since miRNAs are key determinants of EV bioactivity, we used RT-qPCR to detect known MSC-EV miRNAs, as shown in Table 1 [26,35,36]. miR-21-5p, miR-125b and miR-16-5p were all successfully detected in EVs isolated by both EVOs and UC. The same reactions run without EVs (no template control, NTC) showed no amplification (Supplemental Figure S4).

a. Representative cryoEM image of an EV isolated from CCM using EVOs. Scale bar 50 nm; b. Antibody-based membrane array showing EV surface and cargo markers of EVs isolated via EVOs. Fifty-microgram total protein was loaded.

Particle-to-protein ratio of samples from unprocessed CCM, or CCM processed by EVOs, EQ or UC. Unprocessed and EVOs-processed samples were compared by t-test. Each data point represents a separate isolation. *p* values are indicated on the graph.

**Table 1.** Cycle threshold (CT) values for three MSC-EV miRNAs in EVs isolated via EVOs or UC.

miRNA ID	Cycle threshold (CT) ± SD	
	EVOs	UC
miR-21-5p	35.63 ± 0.53	35.51 ± 2.13
miR-125b	34.63 ± 0.49	33.60 ± 0.96
miR-16-5p	35.99 ± 0.87	36.04 ± 1.36
No template (NTC)	Not detected	Not detected

### 3.6. Confirmation of compatibility with downstream testing

Prior to evaluating whether the isolated EVs maintained their functions, we first examined whether the residual PEO (Supplemental Figure S1) could cause cytotoxicity. Testing (Supplemental Figure S5a) showed that up to 20 mg/ml PEO spiked into culture medium had no significant effect on human cardiomyocyte viability. We also measured cell viability under normal culture conditions with unprocessed CCM or EVOs-processed CCM and found no significant difference. Thus, there was no indication that residual PEO affected cell viability which may affect the use of EVOs-EVs in cell-based assays.

### 3.7. Confirmation of EV cardioprotective functional activity

**Figure 6.**
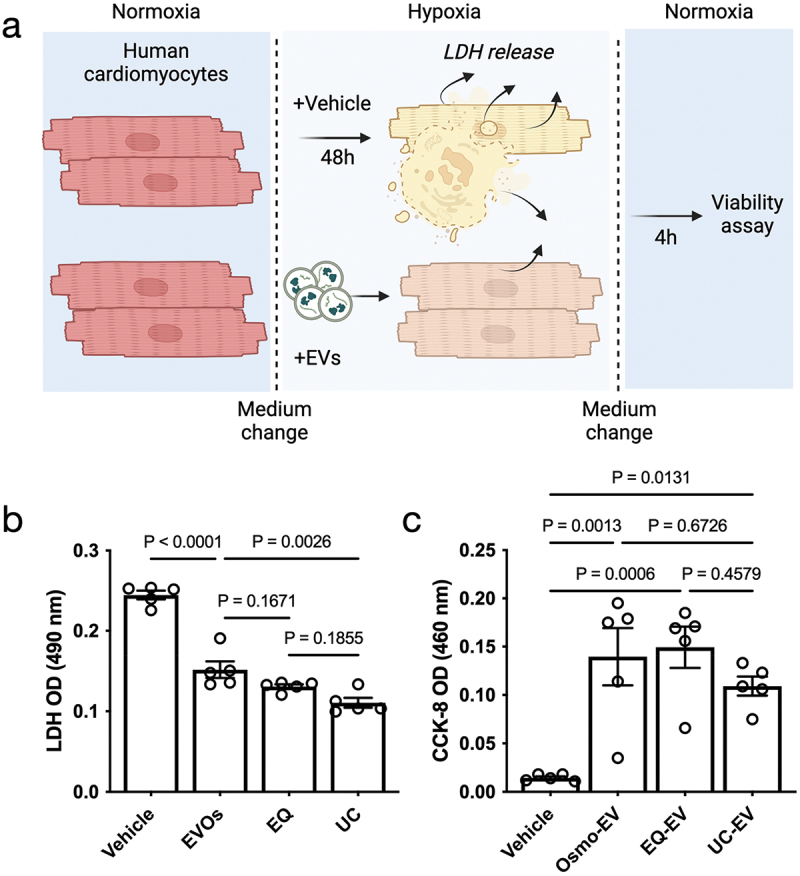
Assessing cardioprotective functionality of extracellular vesicles isolated by EVOs.

MSC-EVs can protect cardiomyocytes from hypoxic injury by delivering their cargo, including miRNAs detected previously. Thus, we sought to evaluate whether EVs isolated by EVOs would preserve those functional protective effects. EVs isolated via EVOs were compared to EVs isolated by EQ and UC provided at the same concentration. Figure 6a shows the timeline and principle of using two assays (LDH and CCK-8) to assess viability. LDH reflects cumulative cell damage over time, whereas CCK-8 measures cellular metabolic activity during the incubation period. As shown in Figure 6b, addition of EVs from all three approaches significantly (*p* < 0.0001) reduced the release of cardiomyocyte LDH compared to the vehicle control group. This shows that all EVs reduced cardiomyocyte damage during hypoxia, confirming their protective functions. The concentration of LDH released by cells treated with EVs from EVOs was not significantly different to EQ (*p* = 0.1671) but was slightly higher than UC-EVs (*p* = 0.0026). CCK-8 viability assay during the re-oxygenation stage is shown in Figure 6c. Here, all EV-treated groups showed significantly higher metabolic activity than vehicle-treated cells (*p* < 0.05) and there was no significant difference in the protective effects of EVs from all three methods. Together, these data show that EVOs could isolate EVs from CCM, preserving their structure, surface and cargo proteins, miRNA cargo, and functional activity.

a. Schematic diagram of experimental design. AC16 human cardiomyocytes were subjected to 48 h hypoxic injury with vehicle control (PBS), EVOs-EVs, precipitation (EQ-EVs) or UC-EVs provided at the same dose; b. LDH released from human cardiomyocytes during 48 h of hypoxia in the presence of vehicle control (PBS), or CCM processed by EVOs, EQ or UC. All groups were compared by one-way ANOVA with Tukey’s post-test; c. Cardiomyocyte viability (CCK-8 assay) after 4-h re-oxygenation injury. All groups were compared by one-way ANOVA with Tukey’s post-test. *p* values are shown on the graphs. Each data point represents an individual sample (n = 5 samples per group).

### 3.8. Using EVOs in combination with size exclusion chromatography

**Figure 7.**
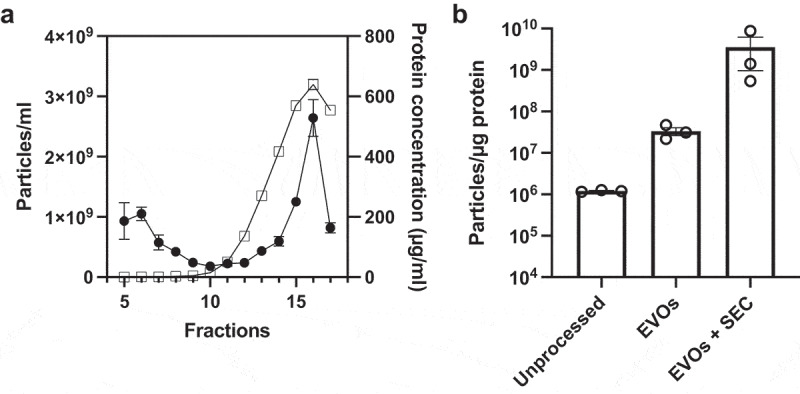
Use of EVOs prior to size exclusion chromatography (SEC).

One limitation of EVOs was the less efficient protein removal, leading to lower EV purity (Figure 5) than EQ or UC. Subjecting the same sample to a second or third EVOs runs with moderately improved EV purity; by 14.58 ± 3.74% and 29.57 ± 3.00%, respectively. However, we reasoned that samples from EVOs could instead be further purified using size-exclusion chromatography (SEC), which is highly effective at removing small proteins [11,21]. Typically, SEC is suboptimal for processing CCM due to the large volumes of medium and relatively low abundance of EVs. However, after EVOs, the CCM volume is reduced by 50-fold and the EVs are more concentrated (Figure 5). Therefore, we subjected EVOs samples to SEC and measured fractions by BCA and NTA (Figure 7a). Using this approach, 30 ml of CCM could be processed using a single run of a 0.5 ml SEC column. The first 4 fractions are column flowthrough, which were discarded. The next several fractions had very low protein concentrations, and free proteins eluted in later fractions, as expected. EV-containing fractions (fractions 5–7) were defined based on the high particle count and low protein concentrations [31,32]. Measuring the particle:protein ratio (Figure 7b) showed that EV purity was significantly increased to 3.6 × 10^9^ particles/µg, which was higher than obtained using UC alone (Figure 5). Together, EVOs enabled SEC to successfully isolate a high concentration of pure EVs from a 50-fold larger volume of CCM in approximately 3 h.

a. Fraction analysis of EVOs-processed CCM samples subjected to SEC. Protein concentration (right Y-axis, open squares) and particle count (left Y-axis, closed circles) are shown for the collected fractions 5–17. Fractions 5–7 were selected as the EV fractions; b. Purity, measured by particles per µg protein, for unprocessed CCM, EVOs-EVs, and EVOs-EVs subsequently processed by SEC.

## 4. Discussion

The EVOs device rapidly isolated EVs from conditioned culture medium of therapeutic cells with minimal user effort, reducing specimen volume and achieving a high yield of EVs. The EVOs device uses size-dependent filtration to separate EVs from smaller particles, driven by osmotic pressure difference rather than centrifugal or hydrostatic forces. Osmosis has some advantages since it directly removes water from the specimen, thereby concentrating substances which are larger than the membrane cut-off. The principle of osmosis-driven filtration was demonstrated in our previous study, which concentrated protein analytes [23]. In the current study, we modified the device for handling cell culture supernatants, aiming to retain particles of ~100 nm while allowing smaller particles such as albumin to pass through the filter. Indeed, our initial approach (Figure 1b) demonstrated >85% albumin removal and ~89% retention of the particles ≥100 nm (Figure 1c). This was also visible by reduction of proteins in SDS-PAGE (Figure 3). Since osmotic pressure is generated by a concentration difference between two sides of a semi-permeable membrane, the use of a highly concentrated water-soluble polymer allowed for rapid mass transport. In this study, we aimed for rapid EV isolation and the PEO concentration was maximized accordingly, allowing the process to be completed in under 2 h.

High research interest in EVs has increased the demand for efficient EV isolation methods, and the field is focused on improving standardization and reproducibility [24]. Current EV purification approaches include tangential flow filtration (TFF), asymmetric flow field-flow fractionation (AF4), ultracentrifugation, precipitation, ultrafiltration, size-exclusion chromatography (SEC), and immunoaffinity capture; each of which has advantages and limitations. Tangential flow filtration (TFF) is increasingly used for industrial EV preparation and shows less filter clogging than conventional dead-end filtration. It also allows for gentle separation, does not introduce reagents into the specimen, and produces concentrated samples. However, the setup and optimization of TFF are much more expensive and time-consuming and it is less commonly used in basic research [37,38]. Asymmetric-flow field-flow fractionation (AF4) has the capability to separate subpopulations of EVs but the input sample volume is limited, which means diluted samples such as conditioned medium must be pre-concentrated by other methods [39,40]. Similar to TFF, AF4 operators need to fine-tune several parameters to achieve optimal separation.

EVs isolated by UC have high purity, but relatively low yields, and the process is time-consuming and less reproducible since it requires manual removal of supernatants and washing/resuspending pellets of EVs. Furthermore, it has been shown that high centrifugal forces can damage or fuse/merge EVs, although this did not disrupt their functional activity in our testing [7,41,42]. On the other hand, precipitation can isolate EVs with very high yields and protocols are relatively simple, although most involve an overnight incubation step. However, the resulting purity of precipitated EVs tends to be lower than UC, which was also shown in our study (Figure 5) [7]. Another disadvantage of precipitation is that the common commercial products use proprietary or undisclosed polymers that are present in the final EV preparation, which may affect biological assays [22]. Chromatography-based methods such as SEC are excellent at removing proteins below the column cutoff size, such as albumin, but are generally less suitable for large volumes of diluted specimens such as CCM, though newer multi-modal SEC offers increased scalability [11,43–45]. SEC inherently dilutes samples which may require concentration using centrifugal filtration columns, resulting in EV loss. Commercial columns are also relatively expensive, have low capacities and can only be used for a limited number of times.

Importantly, there is currently no EV isolation method which is simultaneously rapid, high yield, high purity, and simple. Therefore, the EV isolation methods should be chosen based on the type of specimen, depending on the volume, EV concentration and level of non-EV protein, and the budget and technical expertise of the researchers. For example, UC, UF and TFF are better suited towards processing CCM, urine and other voluminous specimens, whereas SEC is better suited towards plasma, serum, platelet lysates and other concentrated specimens. The EVOs system has been designed to be rapid, simple and achieve high EVs yields from larger sample volumes. Since ultrafiltration membranes may clog with increased processing times or large sample volumes, the EVOs was designed as a single-use device with maximum surface area for filtration to occur. Advantages of the EVOs device are the low labor requirements, simplicity, scalable volume throughput, and processing speed. Our design integrates a 200 μl no-contact reservoir at the bottom of the device where the specimen does not interface with the membrane (Scheme 2b,d). Therefore, the device avoids sample loss, dry-out, and the performance is highly consistent between batches. A user can fill the sample holder with CCM and leave the device until completion, which occurs passively in less than 2 h. UC and precipitation methods require nearly 24-h processing time, which might lead to degradation and/or additional chemical reactions. The long processing time does not match to the typical working hours, which makes the implementation significantly more challenging. EVOs’s rapid processing time, ca. 2 h, allows multiple processes within the 8-h shift. Additionally, the device can be scaled up by running multiple units in parallel. In our study, we also showed that the EVOs system could address the issues associated with SEC by concentrating specimen 50-fold volumetrically, and remove ~97% soluble proteins prior to SEC, which subsequently improved EV purity.

To characterize EVs isolated by EVOs, we followed the latest recommendation from the ISEV [1]. CryoEM images and antibody-membrane array showed that the processed CCM specimens contained particles with intact lipid-bilayer membranes, and we confirmed the presence of EV surface markers such as CD81 and CD63 and cargo markers TSG101 and ALIX. Since EV miRNAs appear to be key drivers of their biological effects, particularly cardioprotection, we confirmed the presence of miR-21, miR-16 and miR-125b (Figure 4 & Table 1) [26,27,36] EVOs-derived EVs demonstrated cardioprotective effects in an *in vitro* cardiomyocyte hypoxia/reoxygenation assay, with their overall therapeutic efficacy equal to the same dose of EVs isolated by UC and EQ (Figure 6). Together, our analysis showed that EVs isolated by EVOs preserved their structural and biological properties. However, the purity of EVs obtained from the standalone EVOs device was lower than comparative methods. This may be due to the choice of filtration membrane where larger pores would improve albumin removal but might lower yields, whereas smaller pores may retain more EVs at the cost of longer processing times. To obtain higher purity EV isolates, EVOs was used as upstream processing to reduce specimen volumes before SEC. This then achieved EV purity higher than UC and made efficient use of the SEC column, processing 30 ml of CCM with a single 0.5 ml column, without the need for subsequent EV concentration. Future work could address the stability and biocompatibility of isolated EVs, as well as validating their therapeutic functions using *in vivo* models.

## 5. Conclusion

In conclusion, an osmosis-driven filtration device, EVOs, was demonstrated as a promising new tool for rapid, high-yield EV isolation from cell-conditioned culture medium. In future, the device could be further validated for its performance in other biological samples. Further optimization of the filtration membrane may also be possible.

## Supplementary Material

Supplemental Material

## Abbreviations

CCK-8: cell-counting kit 8 (a WST-based viability assay)
CCM: cell conditioned culture medium
DI: deionized
EQ: ExoQuick (precipitation-based isolation method)
EV: extracellular vesicle
LDH: lactate dehydrogenase
NTA: nanoparticle tracking analysis
SEC: size exclusion chromatography
TFF: tangential flow filtration
UC: ultracentrifugation
UF: ultrafiltration
