# Supplementary material for: Rapid isolation of extracellular vesicles from stem cell conditioned medium using osmosis-driven filtration
Source: Sci Technol Adv Mater. 2025 Apr 3;26(1):2485668. doi: 10.1080/14686996.2025.2485668 (PMC12001845; doi:10.1080/14686996.2025.2485668)
Supplement: Supplemental Material [file TSTA_A_2485668_SM7387.docx]

**SUPPLEMENTARY INFORMATION**

| **miRNA ID** | **Forward sequence** | **Reverse sequence** |
| --- | --- | --- |
| hsa-miR-21-5p | GCTTATCAGACTGATGTTG | GAACATGTCTGCGTATCTC |
| hsa-miR-16-5p | TAGCAGCACGTAAATATTGG | GAACATGTCTGCGTATCTC |
| hsa-miR-125b | CCTGAGACCCTAACTTG | GAACATGTCTGCGTATCTC |

**Supplementary Table 1.** List of miRNA primers used in this study


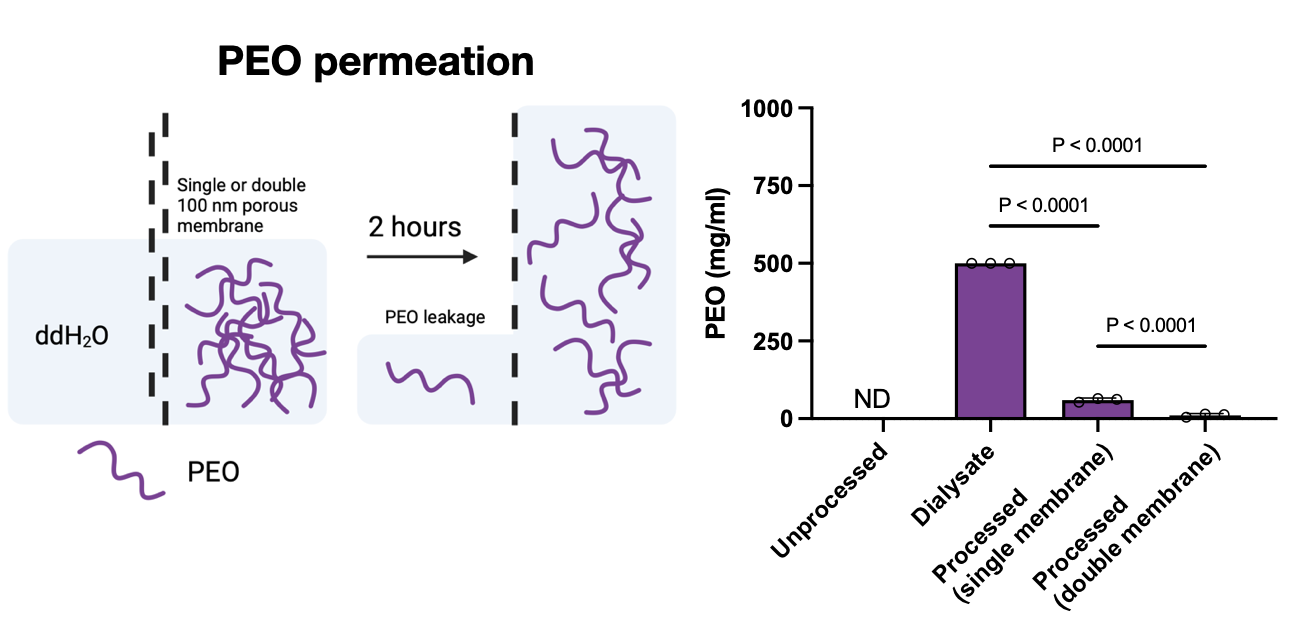


**Supplementary Figure 1. Evaluation of PEO diffusion into specimen**

10 ml ddH_2_O was subjected to osmosis-driven filtration and PEO permeation into the concentrate was measured. The dialysate PEO concentration is 500 mg/ml. Specimens were compared by one-way ANOVA. P values are indicated on the graph.

**Supplementary Figure 2. Osmolarity measurements for PEO solutions.**

PEO solutions with 1, 2, 4, and 8 wt% in DI water were used for the osmolarity measurements. The 2^nd^ order polynomial fitting result (R^2^ = 0.9967) was

Osmolarity = 0.8889 × (Polymer concentration)^2^ + 2.119 × Polymer concentration + 0.7724.


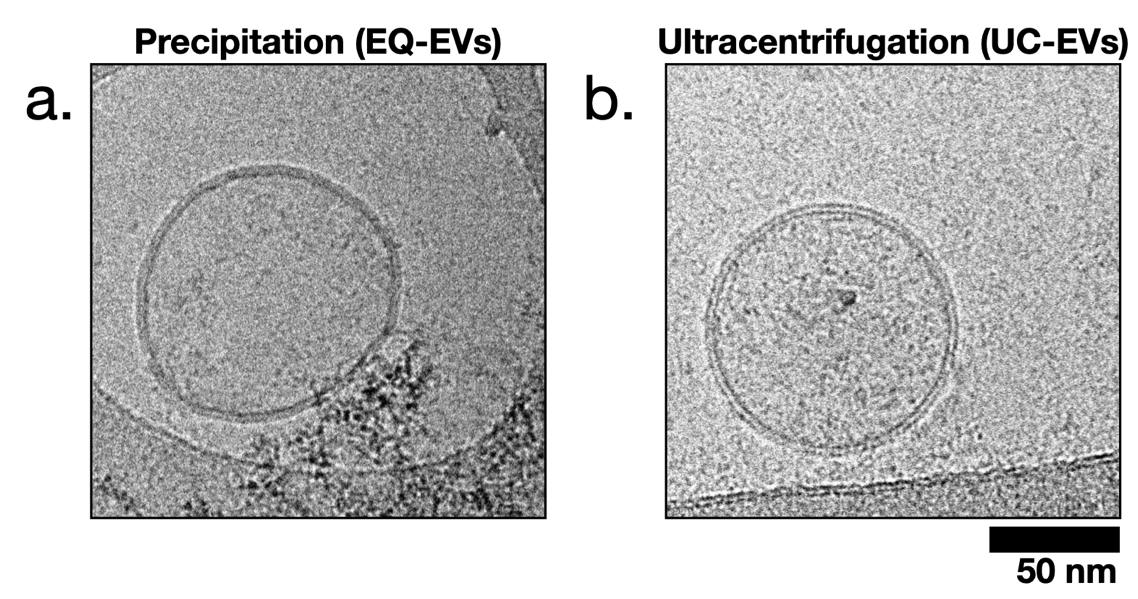


**Supplementary Figure 3. CryoEM of EVs isolated by EQ and UC**

1. cryoEM image of EVs isolated using precipitation (ExoQuick-TC, EQ)
2. cryoEM image of EVs isolated using ultracentrifugation (UC-EVs)

Scale bar of 50 nm is indicated


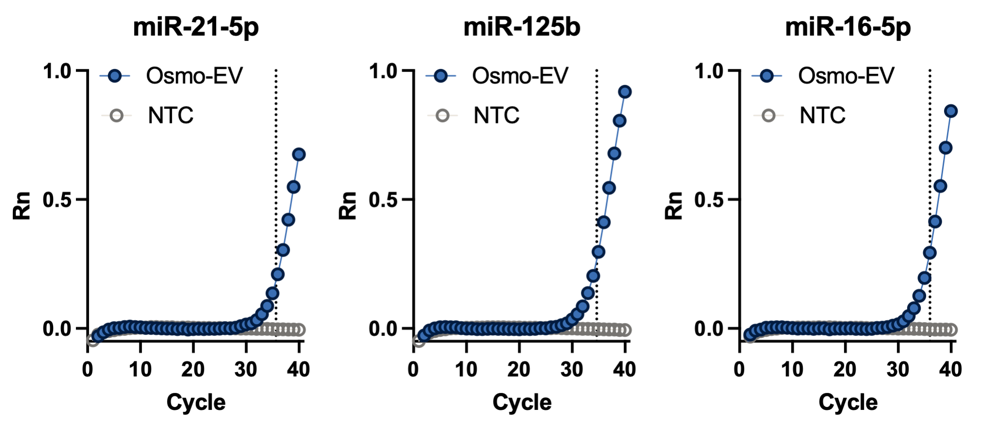


**Supplemental Figure 4. Amplification curves of Osmo-EV miRNA cargo**

Amplification plots for known abundant miRNAs in MSC EVs isolated by osmosis. Samples without template (NTC) were used as a negative control. The Y axis shows normalised fluorescence signal Rn for cycles 1 - 40. The dotted line on the X axis indicates the cycle threshold (CT) value.


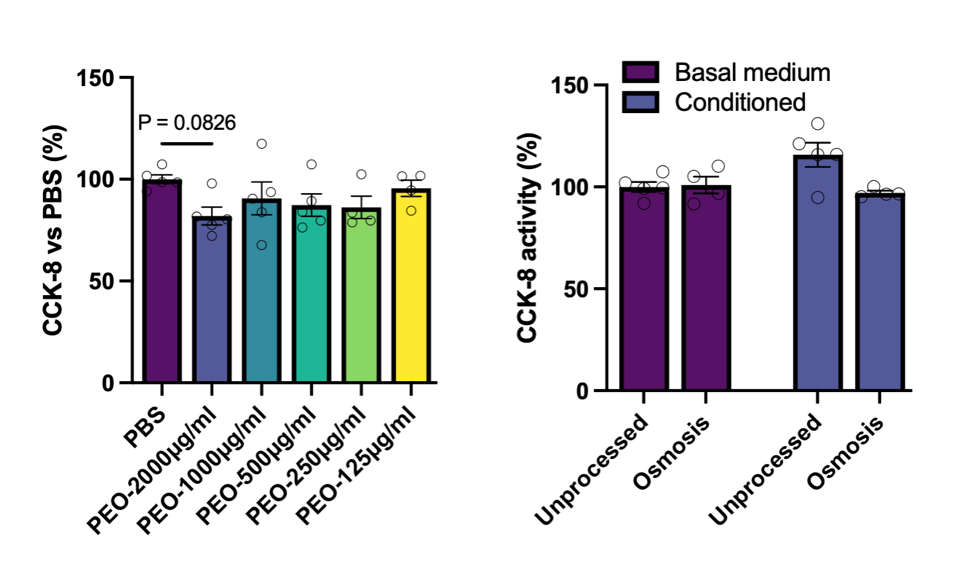


**Supplementary Figure 5. Evaluation Potential Toxicity of Residual PEO**

1. Cell viability analysis after 48h incubation with spike-in PEO which was added at 10% (v/v) to form final concentrations of 125 to 2,000 µg/ml. All samples were compared to spiked PBS (10% v/v) by one-way ANOVA. The P value compared to the highest dose is shown. No significant reduction in viability was observed in any group.
2. Cell viability following 48h incubation with basal medium or CCM which was unprocessed or concentrated by osmosis. Samples were compared by paired *t*-test. No significant differences were observed.
